# Are Patients with Erythema Migrans Who Have Leukopenia and/or Thrombocytopenia Coinfected with *Anaplasma phagocytophilum* or Tick-Borne Encephalitis Virus?

**DOI:** 10.1371/journal.pone.0103188

**Published:** 2014-07-24

**Authors:** Franc Strle, Petra Bogovič, Jože Cimperman, Vera Maraspin, Katarina Ogrinc, Tereza Rojko, Daša Stupica, Lara Lusa, Tatjana Avšič-Županc, Katja Strašek Smrdel, Mateja Jelovšek, Stanka Lotrič-Furlan

**Affiliations:** 1 Department of Infectious Diseases, University Medical Center Ljubljana, Ljubljana, Slovenia; 2 Institute for Biostatistics and Medical Informatics, Faculty of Medicine Ljubljana, Ljubljana, Slovenia; 3 Institute of Microbiology and Immunology, Faculty of Medicine Ljubljana, Ljubljana, Slovenia; University of Texas Medical Branch, United States of America

## Abstract

Lyme borreliosis (LB), tick-borne encephalitis (TBE) and human granulocytic anaplasmosis (HGA) are endemic in central part of Slovenia. We tested the hypothesis that patients with erythema migrans (EM) from this region, who have leukopenia and/or thrombocytopenia (typical findings in HGA and in the initial phase of TBE but not in patients with LB) are coinfected with *Anaplasma phagocytophilum* and/or with TBE virus, i.e. that cytopenia is a result of concomitant HGA or the initial phase of TBE. Comparison of clinical and laboratory findings for 67 patients with EM who disclosed leukopenia/thrombocytopenia with the corresponding results in sex- and age-matched patients with EM and normal blood cell counts revealed no differences. In addition, patients with typical EM and leukopenia and/or thrombocytopenia tested negative for the presence of IgM and IgG antibodies to TBE virus by ELISA as well as for the presence of specific IgG antibodies to *A. phagocytophilum* antigens by IFA in acute and convalescent serum samples. Thus, none of 67 patients (95% CI: 0 to 5.3%) with typical EM (the presence of this skin lesion attests for early Lyme borreliosis and is the evidence for a recent tick bite) was found to be coinfected with *A. phagocytophilum* or had a recent primary infection with TBE virus. The findings in the present study indicate that in Slovenia, and probably in other European countries endemic for LB, TBE and HGA, patients with early LB are rarely coinfected with the other tick-transmitted agents.

## Introduction

Hard ticks transmit etiologic agents of several human and animal diseases. In the large part of Europe the main tick vector is *Ixodes ricinus* and the principal agents are Lyme borreliae (causing Lyme borreliosis, LB), *Anaplasma phagocytophilum* (the causative agent of human granulocytic anaplasmosis, HGA) and tick-borne encephalitis virus (causing tick-borne encephalitis, TBE). Reports on coinfections with several microorganisms and coexistence of the corresponding diseases in humans are rather limited. In the USA studies reported simultaneous LB and HGA [1]–[4] and a few reports deal with concurrent LB and babesiosis [5]–[7] while in Europe cases of simultaneous LB and HGA [8]–[13], concomitant LB and TBE [8], [14]–[16], as well as concurrent HGA and TBE have been published [8], [13], [17]. However, reported coinfection rates and clinical characteristics of coinfections are quite heterogeneous as they depend upon several factors – as pointed out more than 10 years ago [8] and recently [4] definitions used for diagnosis substantially influence the appreciation of coinfection rates as well as interpretation of illness severity.

In Slovenia, a small Central European country with 2 million inhabitants, LB, TBE and HGA are endemic. Mandatory notification exists for TBE (from 1976), for LB (from 1986) but not for HGA. The most frequent tick-borne disease in humans is LB (during the last few years incidence rates were between 244–309 cases/100.000 inhabitants) followed by TBE (8–15 cases/100.000) [18] and HGA (up to 0.5 cases of proven HGA/100.000 inhabitants per year). The main and by far the most frequent manifestation of LB is erythema migrans (EM), a skin lesion which develops at the site of inoculation of borreliae by the bite of infected tick within 1 month after the bite. In Europe the lesion is accompanied by mild local symptoms in about 50% while systemic symptoms are present in approximately 1/3 of patients. In adult patients fever is present only exceptionally [19], [20]. In contrast, TBE is a febrile illness, which also develops within 1 month after a tick bite and has typically a biphasic course. The initial phase, which corresponds to viremia and lasts for 1–8 days, is characterized by fever, headache and malaise. It is followed by an improvement or even an asymptomatic interval of about one week, and then by signs and symptoms of neurological involvement. The second phase presents as meningitis, meningoencephalitis, or meningoencephalomyelitis in 50%, 40%, and 5–10% of adult patients, respectively [21]. The initial phase of TBE is quite a challenge for a proper diagnosis because clinical features are unspecific and antibodies to TBE virus are as a rule absent. However, the initial phase of TBE (but not the second phase of the disease) is often associated with leukopenia and/or thrombocytopenia. For example, in one of the reports bicytopenia was established in 71.4% (20/28) patients, only leukopenia in one (3.6%) patient while in three (10.7%) patients thrombocytopenia without leukopenia was recorded; in addition in 22.2% of patients mild abnormalities of liver enzymes were found [22]. HGA also develops within <1 month after a tick bite and usually consists of fever, headache, malaise, myalgia and/or arthralgia. Similarly to the initial phase of TBE, laboratory findings of leukopenia, thrombocytopenia, and elevated liver enzymes are often present [23]. In contrast, patients with EM have their blood cell counts as a rule in a normal range. In adult European patients with EM leukopenia has been reported to be present in 10.1%, thrombocytopenia in 1.8% and mildly abnormal liver function tests results in 10.9% of patients [24].

The aim of our study was to test the hypothesis that patients with EM from central part of Slovenia, where LB, TBE as well as HGA are endemic, who have leukopenia and/or thrombocytopenia are coinfected with *A. phagocytophilum* and/or with TBE virus, i.e. that cytopenia is a result of concomitant HGA or the initial phase of TBE.

## Patients and Methods

### Patients

In the period 2006–2012, 3443 patients were diagnosed with EM according to European case definition criteria [25] at the Outpatient’s Lyme borreliosis Clinic of the University Medical Center Ljubljana, Slovenia and in 3133 (91%) basic laboratory tests were performed. Of them 122 (3.9%) had leukopenia (leukocyte count <4×10^9^/L) and/or thrombocytopenia (platelet count <140×10^9^/L) at the initial examination. These 122 patients were eligible for the present study since they did not have a known underlying condition associated with low blood cell counts (such as cirrhosis, excessive alcohol consumption, aplastic anemia, systemic lupus erythematosus, myelofibrosis, Hodgkin lymphoma, cancer, etc) and they were not receiving chemotherapy, radiation therapy, immunotherapy, or antiepileptic treatment; of 122 patients with leukopenia and/or thrombocytopenia 67 (54.9%) patients for whom serum specimens obtained at enrollment and 14 days (or up to 2 months) later were available, were included in the study.

In all patients with EM a structured interview that comprised demographic data, detailed information on tick bites, EM and potential other manifestations of LB, questions about 9 particular systemic symptoms, including fever, headache, myalgia and arthralgia, questions about 3 symptoms at the site of EM lesion (burning, itching, pain), and a physical examination were performed at the initial visit. Laboratory studies included a complete blood cell count with differential and platelet count, liver function tests, and serologic testing for the presence of IgM and IgG antibodies to *Borrelia burgdorferi* sensu lato. In addition, 2 ml serum was obtained at first visit and 14 days (or up to 2 months) later; the specimens were frozen at –20°C or at –80°C for potential further testing.

The approach was approved by the Medical Ethics Committee of the Republic of Slovenia (No 38/05/06 and No 36/05/09). Written informed consent was obtained from all participants. The investigation was conducted according to the principles expressed in the Declaration of Helsinki. In addition, the present study, which is based on the material obtained previously, was also approved by the Medical Ethics Committee of the Republic of Slovenia (No 98/03/14).

### Diagnostic evaluation for coinfection with *Anaplasma phagocytophilum* and tick-borne encephalitis virus

Serum antibodies to *A. phagocytophilum* and to TBE virus were determined in paired serum specimens obtained at the first visit due to EM and 14 days later (in a few patients up to 2 months after the initial sample). The analyses were performed in June 2013; thus, specimens were kept stored for 7 months to up to 7 years and 4 months prior to testing.

The presence of serum IgM and IgG antibodies against TBE virus was determined by ELISA (Enzygnost Anti-TBE/FSME virus). According to the manufacturer instructions, TBE IgG values above 5.0 U/mL were considered as a positive result; TBE IgM values above 0.372 were interpreted as positive and TBE IgM values from 0.272 to 0.372 as borderline (Siemens, Marburg, Germany) [26].

In addition, acute and convalescent serum samples were tested by immunofluorescent assay (IFA) for the presence of specific IgG antibodies to *A. phagocytophilum* antigens as previously described. Endpoint titers were recorded as the reciprocal of the last serial dilution at which specific apple-green fluorescence of ehrlichial inclusion bodies was focally located in the cytoplasm of the infected cells. Reciprocal IFA titers of ≥1∶256 to *A. phagocytophilum* were interpreted as a positive result [23].

### Comparison of clinical and laboratory findings

Clinical and laboratory findings of patients with EM who disclosed leukopenia/thrombocytopenia were compared with the corresponding findings in the control group of patients with EM and normal blood cell counts. The control group consisted of patients who were diagnosed with EM at our Lyme borreliosis Outpatient Clinic in the same year and were matched for sex and age. When several control patients comply with all three criteria the patient with last name alphabetically the most similar to the corresponding index patient was chosen as a control.

In addition, comparison of clinical and laboratory findings was performed also within the group with leukopenia/thrombocytopenia, i.e. between patients in whom coinfection was established and with those in whom it was not.

### Statistical analysis

Categorical data were analysed with Yates’ corrected chi-square or Fishers’ exact test (2-tailed), and numerical data were analysed by the Kruskal-Wallis test, using Epi info version 3.4.3 software (Centers for Disease Control, Atlanta, Ga). To take into account multiple testings a *P* value <0.01 was considered to be significant. Categorical data were summarized with percentages 95% exact binomial confidence intervals.

## Results

In the period of seven years 122/3133 (3.9%) patients diagnosed with EM had leukopenia and/or thrombocytopenia at the initial visit. Paired serum specimens were available for 67/122 patients (54.9%). Leukopenia was present in 37/67 (55.2%, 95% CI: 42.6 to 67.4%) patients (median 3.7, range 0.3–3.9×10^9^/L), thrombocytopenia was found in 32/67 (47.8%, 95% CI: 35.4 to 60.3%) patients (median 132, range 75–139×10^9^/L); only three patients (4.4%, 95% CI: 1 to 12.5%) had bicytopenia (leukopenia and thrombocytopenia). All 134 paired acute and convalescent serum specimens on 67 patients (0%, 95% CI: 0 to 5.3%) were negative for antibodies to *A. phagocytophilum*. In none of the 134 serum samples IgM antibodies to TBE virus were detected, however 9 (13.4%, 95% CI: 6.3 to 24.0%) patients had IgG antibodies to the virus present in paired specimens. In all 9 patients history on previous TBE was negative. Six (66.7%) of them had been vaccinated for TBE, and all denied having had yellow fever or received yellow fever vaccine which might have resulted in positive IgG antibody findings. In 6/9 (66.7%) patients antibody levels in the paired samples were comparable while in three substantial increase was detected comparing the initial and »convalescent« sample (2, 2, and 8 weeks later, respectively). In the group of patients with antibody increase two patients had been completely vaccinated against TBE (the last booster doses were obtained 2 and 5 years prior to current testing, respectively) while the third patient had not received the vaccine.

Patients with EM and leukopenia/thrombocytopenia did not differ from patients with EM without leukopenia/thrombocytopenia according to the duration of EM skin lesion (as noticed by patient) prior to diagnosis, the largest diameter of EM, frequency of associated systemic symptoms (including fever, headache, myalgia and arthralgia) and abnormal findings on physical examination as well as proportion of those with abnormal liver function test results (Table 1).

**Table 1:** Characteristics of patients with erythema migrans (EM) with associated leukopenia and/or thrombocytopenia, and patients with EM with normal blood cell count (controls).

Characteristics	Patients with EM and		*P* value
	leukopenia and/or thrombocytopenia (n = 67)	normal cell counts (n = 67)	
Age, years; median (range)	54 (18–83)	52 (17–81)	0.7150
Male sex	36 (53.7%)	36 (53.7%)	>0.99
Tick bite	37 (55.2%)	42 (62.7%)	0.4824
>1 tick bite	7 (10.4%)	10 (14.9%)	0.6037
Days since tick bite to EM; median (range)^a^	14 (2–90)	15.5 (2–90)	0.4992
Multiple EM	5 (7.5%)	8 (11.9%)	0.5594
Largest diameter of EM, cm; median (range)	12 (5–42)	13 (5–50)	0.3747
Duration of EM, days; median (range)^b^	7 (2–45)	7 (1–90)	0.0349
arthralgias	1 (1.5%)	5 (7.5%)	0.2081
Leukocyte count <4×10^9^/L	37 (55.2%)^c^	0	
median (range)	3.7 (0.3–3.9)		
Platelet count <140×10^9^/L	32 (47.8)^d^	0	
median (range)	132 (75–139)		
Elevated ESR (>20 mm/h)	6/52 (11.5%)	8/55 (14.5%)	0.2571
Elevated AST (>0.58 µkat/L)	9/67 (13.4%)	7/67 (10.4%)	0.7899
Elevated ALT (>0.74µkat/L)	14/67 (20.9%)	6/67 (9%)	0.0897

EM, erythema migrans; ESR, erythrocyte sedimentation rate; AST, aspartate aminotransferase enzyme; ALT, alanine aminotransferase enzyme; TBE, tick-borne encephalitis.

a Calculated for patients with one tick bite.

b Duration of erythema migrans (as noticed by patient) prior to diagnosis.

c Of 37 patients with leukopenia at the initial visit, 6 (16.2%) also had decreased blood lukocyte counts at the time of convalescet serum sampling.

d Of 32 patients with thrombocytopenia at the initial visit, 8 (25%) also had decreased blood platelet counts at the time of convalescet serum sampling.

As expected, within the group of patients with leukopenia/thrombocytopenia, no difference in epidemiological, clinical and laboratory findings was found comparing patients with IgG antibodies to TBE virus in serum with those who had no serological indication for the contact with TBEV.

## Discussion

The main stimulus for our study was limited information on coinfections in scientific literature. The coinfection issue is not merely of academic interest but is also pertinent to the real-life occurrences. Despite scarce scientific information, search for coinfections is performed more and more often in patients with LB.

Slovenia is a highly endemic region for LB and TBE and is the country with the highest number of reported HGA cases in Europe. The etiologic agents of the three illnesses are transmitted by the bite of the same vector tick (*I. ricinus*). In Slovenia, Lyme borreliae were found in 13% of examined nymphs and in 22–35% of adult ticks [27], while infection rates of *I. ricinus* ticks with TBE virus and *A. phagocytophilum* were 0.4%–0.5%, and 3.2%, respectively [28], [29]. The data on tick infection rates and reports on simultaneous LB and HGA [8]–[13], concomitant LB and TBE [8], [14]–[16] and concurrent HGA and TBE [8], [13], [17] were the basis for our assumption that some patients with EM, the early localized form of LB, could be coinfected with TBE virus and/or *A. phagocytophilum*. Since leukopenia and thrombocytopenia are rather characteristic laboratory findings for the initial phase of TBE as well as for HGA while patients with LB have as a rule the blood cell counts within normal range, we hypothesized that the chances for detection of a coinfection are the highest in patients with EM associated with leukopenia and/or thrombocytopenia.

The results of the present study did not support our hypothesis. Leukopenia and thrombocytopenia were rare and were only exceptionally present in combination, and the clinical characteristics of patients with EM and associated cytopenia were rather ordinary. No relevant distinction in clinical characteristics was uncovered comparing patient with EM and leukopenia/thrombocytopenia with age- and sex-matched patients who had EM but normal blood cell counts (Table 1). Furthermore, in none of the 67 patients with typical EM and associated leukopenia and/or thrombocytopenia antibodies to *A. phagocytophilum* were established, and in none of them (95% CI: 0 to 5.3%C) recent infection with TBE virus (indicated by the presence of specific IgM antibodies) was ascertained. However, in 9/67 (13.4%) patients the presence of IgG antibodies to TBE virus in the first and the convalescent serum specimens was established. Such finding points to previous (symptomatic or asymptomatic) infection with TBE virus, vaccination against TBE, or previous infection with or vaccination against viruses similar to TBE virus, such as yellow fever virus. In our group of nine patients explanation for the presence of TBE IgG antibodies was rather straightforward for six patients who had been vaccinated against TBE. The remaining three patients most probably had had asymptomatic infection with TBE virus in past since none of them recalled having had TBE nor had received yellow fever vaccine that might have resulted in similar serological result. In 6/9 patients the specific antibody levels in paired samples were similar while in three patients substantial increase was established comparing the first and the second sample antibody levels, indicating the possibility of a buster phenomenon. Since none of the patients received TBE vaccine within the last 6 months, since all our patients had a typical EM skin lesion and since the presence of the skin lesion attests for a recent tick bite, the increase in TBE virus IgG antibody levels was probably associated with a recent bite of the TBE virus-infected tick.

Our microbiological approaches for the detection of infections with TBE virus and *A. phagocytophilum* were limited to serological testing of paired serum samples, obtained at the time of diagnosis of EM (median 21 days after a tick bite) and 14 days to 2 months later. The absence of antibody response 3 and 5–11 weeks after exposure rather reliably exclude an infection with the corresponding agents. Nevertheless, although not reported previously, there is a theoretical possibility that antibiotic treatment for EM abrogated antibody response to *A. phagocytophilum.* If valid, this could have been an explanation for at most 33 patients (49.3%), that is for those who were treated with doxycycline – a drug of choice for therapy of EM as well as of HGA – but could not apply for the other half of our patients with EM who received antimicrobial agents (such as amoxicillin, cefuroxime or azithromycin) that are not active against *A. phagocytophilum*. No such explanation is valid for antibody response to TBE virus.

Leukopenia and/or thrombocytopenia were detected in a small proportion (3.9%) of our patients with EM. Given that the search for coinfection with TBE virus and *A. phagocytophilum* did not reveal positive results, the explanation for decreased blood leukocyte and platelet counts remained unclear. Leukopenia and/or thrombocytopenia might be the result of unrecognized underlying chronic illness (in spite of the fact that patients with known chronic illness and medications associated with decreased blood cell counts were excluded), coinfection with some other agent associated with cytopenia that we did not test for (such as *Babesia*), or might theoretically be associated with Borrelia infection itself. Considering that of the 37 patients who had leukopenia and 32 patients who had thrombocytopenia at the initial visit, only 6 (16.2%) and 8 (25%) patients, respectively, had the corresponding cytopenias at the time of convalescent serum sampling (Table 1), the presumption of a chronic underlying illness as a major cause of the decreased blood cell counts seems rather unlikely. However, normal limits for blood cell counts are determined by the 2.5^th^ lower and upper percentile, so values outside this range do not necessarily indicate disease.

In conclusion, none of 67 patients (95% CI: 0 to 5.3%) with typical EM (the presence of this skin lesion attests for early LB and is the evidence for a recent tick bite) associated with mild leukopenia and/or thrombocytopenia was found to be coinfected with *A. phagocytophilum* or had a recent primary infection with TBE virus. Thus, our findings indicate that in Slovenia, and probably in other European countries endemic for LB, TBE and HGA, patients with early LB are rarely coinfected with the other tick-transmitted agents.
